# Notes and News

**Published:** 1903-08-01

**Authors:** 


### NOTES AND NEWS.

The Royal Victoria Hospital, which was opened by the King and Queen during their visit to Belfast on Monday, serves to commemorate Queen Victoria's Jubilee. It contains 300 beds. The hospital, designed by Mr. William Henman, forms the most complete example of an institution arranged for the Plenum system of artificial ventilation. The planning is quite original, based on an attempt to economise labour and concentrate the wards as much as possible. The experiment embodied in the buildings cannot fail to be interesting, for if the Plenum system does not prove a complete success under such favourable conditions as prevail at Belfast, it is to be hoped that it will never be tried elsewhere in the British islands. The plans and a description were published in The Hospital of June 22nd, 1901.

The Duchess of Albany has become patroness of the Oxford Eye Hospital. When being conducted through the wards on the 23rd ult., H.R.H. mentioned that hospitals always aroused her warm sympathies, but it had so chanced that she had never before visited one devoted to the treatment of eye complaints only.

Miss E. Hunt, of Sutton, has bequeathed £1,000 to the Evelina Hospital, to the Middlesex Hospital £200, and £100 to the Samaritan Free Hospital.

The German Government recently refused to sanction the establishment of a Chair of Homœopathy at the University of Tübingen.

The whole truth of a report which reaches us from Paris has yet to be learnt. It is stated that the well-known journalist, Mr. B. Marriott, has been detained in the Insane Asylum at Charenton for fifty-seven days. Mr. Marriott is bringing an action against the physician upon whose certificate he was incarcerated and claims 50,000 francs damages. He affirms that he was detained owing to malicious and false reports, and was only released after continuous endeavours on the part of the British Consulate.

The Chelsea Hospital for Women has received a bequest of £500 from the estate of the late Mr. Richard Bishop, allotted at the discretion of the executors per Dr. Fenton.

That cleanliness is next to godliness appears to be largely recognised by the clergy nowadays. The Church has its Sanitary Association, and this society has appealed to the clergy generally to preach the gospel of health from the pulpits on a special Sunday in the year.

Mr. Perks asked a question in the House of Commons last week in regard to experiments on animals which entailed enforced overfeeding, without anæsthetics. Although animals utilised for the advancement of science benefiting humanity find numerous protectors, we are not aware that anyone has yet risen in Parliament to question the practice of enforced overfeeding of fowls for the epicure's table.

The notice which appears in the *Daily Mail* of the 29th ult. to the effect that the Committee of King's College Hospital have decided in favour of moving that institution into a less congested district of the metropolis will be welcomed by everyone who takes a broad view of the most important question affecting our voluntary hospitals at the present time. Apparently, judging from the notice referred to, the medical staff was unanimously in favour of removal. If such unanimity were to be followed by the medical staffs of other institutions whose removal has been meditated, many of the difficulties in the way of a satisfactory decision would disappear.

Westminster is about to organise a local health society. Associations of the kind are much needed in the metropolis, for the question of sanitation affects every area, but to secure effective interest it is necessary that the object lessons should be local. The housing question and the feeding of infants are of foremost importance. It may take a very long time to teach the necessary lessons, but reiterated precept tells at last in practice. The recently published accounts of the London County Council in connection with their dwellings show profits which should encourage similar undertakings, as they prove that workpeople can be properly housed without loss and in some cases with substantial profit.

Even if the proposals of Colonel Sadler in the House of Commons were carried into effect that an order should be issued empowering local authorities to isolate small-pox contacts during the period of incubation, or alternatively that they should be vaccinated or revaccinated, there would still remain enough possibility of the spread of the disease. Colonel Sadler's further proposal that false or suppressed information should be made punishable is a course necessary, but is already in force. A case has just occurred at Barking where a labourer, who pleaded ignorance, has been sent to prison for failing to notify the fact that his wife was suffering from small-pox. Five cases have already resulted from this negligence, and even where negligence is apparently not present, as in the cases at Cambridge, the difficulties of diagnosis come in. All this is a strong plea for the preventive measure of vaccination.

